# Dynamics analysis of a nonlocal diffusion dengue model

**DOI:** 10.1038/s41598-023-42440-3

**Published:** 2023-09-14

**Authors:** Kangkang Chang, Zhenyu Zhang, Guizhen Liang

**Affiliations:** 1 School of Mathematics and Statistics, Xinxiang University, Xinxiang, 453003 People’s Republic of China; 2 Academy of Fine Arts, Xinxiang University, Xinxiang, 453003 People’s Republic of China

**Keywords:** Applied mathematics, Viral infection

## Abstract

Due to the unrestricted movement of humans over a wide area, it is important to understand how individuals move between non-adjacent locations in space. In this research, we introduce a nonlocal diffusion introduce for dengue, which is driven by integral operators. First, we use the semigroup theory and continuously Fr*é*chet differentiable to demonstrate the existence, uniqueness, positivity and boundedness of the solution. Next, the global stability and uniform persistence of the system are proved by analyzing the eigenvalue problem of the nonlocal diffusion term. To achieve this, the Lyapunov function is derived and the comparison principle is applied. Finally, numerical simulations are carried out to validate the results of the theorem, and it is revealed that controlling the disease’s spread can be achieved by implementing measures to reduce the transmission of the virus through infected humans and mosquitoes.

## Introduction

Dengue fever is severe vector-borne infectious disease transmitted by mosquitoes carrying the dengue virus. Outbreaks arise in various countries annually, posing a significant challenge to global public health. Mathematical models have become invaluable tools for grasping the transmission dynamics and behavior of diseases^1–11^. For instance, Li et al.^1^ explored a reaction-diffusion dengue model that incorporated both wild and Wolbachia-infected mosquito populations, assessing their dynamics and control measures. Xu and Zhao^3^ devised a model for dengue transmission, examining the stability of both disease-free and endemic states. Zhu et al.^5^ introduced a dengue model with a free boundary and derived conditions under which the disease either disappears or spreads. Similarly, Fang et al.^7^ took into account a time-space periodic environment, determining the correlation between the speeds of almost pulsating waves and disease spread. Chang et al.^9^ crafted a diffusion model for dengue influenced by lévy noise, outlining the conditions for near-optimal controls. Zhu et al.^11^ evaluated a dengue model with nonlocal incidence and free boundaries, ascertaining conditions for the disease’s disappearance or proliferation.

A critical observation from the above literature is their reliance on reaction-diffusion models, where the Laplace operator essentially governs the localized random diffusion behavior at nearby spatial locations. However, human movement is not always limited to adjacent areas, indicating that the Laplacian operator based reaction-diffusion model might fall short in depicting long range disease transmission effects^12^. For a more accurate representation of movement between distant locations, our study introduces nonlocal diffusion, where the diffusion process is characterized using integral operators ($\int _{\Omega }{\mathcal {J}}(x-y)\varphi (y)dy-\varphi (x)$)^13^, where ${\mathcal {J}}(\cdot )$ is an even function with probability density one, ${\mathcal {J}}(x-y)$ represents the probability of jumping from position *y* to position *x* and ${\mathcal {J}}(x-y)\varphi (y)dy$ denotes the rate at which individuals reach the position *x* from other positions. For more on nonlocal diffusion, readers can refer to existing literature^14–20^. The main objectives of this study are: (1) constructing a nonlocal diffusion dengue model. Using the Fréchet differentiability and semigroups theory, we validate the solution’s existence, uniqueness, and boundedness. (2) Using the eigenvalue problem of the nonlocal diffusion term and constructing a Lyapunov function, we prove the model’s global stability and uniform persistence. (3) Through numerical simulations, we discuss the global stability and consistent persistence of the disease are. When the disease persists, we analyze the diffusion impact on infected humans and mosquitoes.

This study unfolds as follows: In “Model and preliminaries” section, we present the model and subsequently prove the existence, uniqueness, positiveity, and boundedness of solutions. Using the next-generation operator, we define the basic reproduction number. “Global stability and uniform persistence” section focuses on proving the global asymptotic stability and uniform persistence of the system, achieved through the construction of Lyapunov functions and the application of the comparison principle. “Numerical simulations” section provides numerical simulations. Finally, “Conclusions” section concludes the article.

## Model and preliminaries

To assess the impact of nonlocal diffusion on the dengue model, we begin by introducing the SIR-SI model detailed in^21^, the parameters are defined in Table 1.

1 $$\begin{aligned} {\left\{ \begin{array}{ll} \frac{d S_{H}}{dt}=\mu _{h}N_{H}-\mu S_{H}-\frac{\beta _{H}b}{N_{H}+m}S_{H}I_{V},\\ \frac{d I_{H}}{dt}=\frac{\beta _{H}b}{N_{H}+m}S_{H}I_{V}-(\mu +\gamma _{H})I_{H}, \\ \frac{d R_{H}}{dt}=\gamma _{H}I_{H}-\mu R_{H},\\ \frac{d S_{V}}{dt}=A-\nu S_{V}-\frac{\beta _{V}b}{N_{H}+m}S_{V}I_{H},\\ \frac{d I_{V}}{dt}=\frac{\beta _{V}b}{N_{H}+m}S_{V}I_{H}-\nu I_{V}, \\ \end{array}\right. } \end{aligned}$$

**Table 1:** Definitions of all parameters.

Parameters	Description
$\mu _{h}$	The birth rate of human
$N_{H}$	The population of human
$\mu$	Natural death rate of human
$\beta _{H}$	The transmission rate of dengue to the human from the mosquito
*b*	The mosquitoes biting rate
$\gamma _{H}$	The recovery rate of human
*A*	The recruitment rate of mosquitoes
$\nu$	The nature death rate of mosquitoes
$\beta _{V}$	The transmission rate of dengue to the mosquito from human
*m*	The densities of alternative hosts

It’s worth noting that mosquitoes generally have a limited, activity range, typically flying only tens to hundreds of meters. The furthest recorded flight distance is one to two kilometers. Given this, the nonlocal spread of mosquitoes was disregarded. Also, since the third equation doesn’t feature in the other equations of system (1), we focus on the subsequent dengue model:

2 $$\begin{aligned} {\left\{ \begin{array}{ll} \frac{\partial S_{H}}{\partial t}=d_{1}\int _{\Omega }{\mathcal {J}}(x-y)S_{H}(y,t)dy-d_{1}S_{H}(x,t)+\mu _{h}(x)N_{H}-\mu (x) S_{H}(x,t)-\frac{\beta _{H}(x)b(x)}{N_{H}+m}S_{H}(x,t)I_{V}(x,t),\\ \frac{\partial I_{H}}{\partial t}=d_{2}\int _{\Omega }{\mathcal {J}}(x-y)I_{H}(y,t)dy-d_{2}I_{H}(x,t)+\frac{\beta _{H}(x)b(x)}{N_{H}+m}S_{H}(x,t)I_{V}(x,t)-(\mu (x)+\gamma _{H}(x))I_{H}(x,t), \\ \frac{\partial S_{V}}{\partial t}=A(x)-\nu (x) S_{V}(x,t)-\frac{\beta _{V}(x)b(x)}{N_{H}+m}S_{V}(x,t)I_{H}(x,t),\\ \frac{\partial I_{V}}{\partial t}=\frac{\beta _{V}(x)b(x)}{N_{H}+m}S_{V}(x,t)I_{H}(x,t)-\nu (x) I_{V}(x,t),\\ S_{H}(x,0)=S_{H,0}(x), I_{H}(x,0)=I_{H,0}(x), S_{V}(x,0)=S_{V,0}(x) I_{V}(x,0)=I_{V,0}(x),\\ x\in \Omega ,~~t>0, \\ \end{array}\right. } \end{aligned}$$

with Neumann boundary condition (the derivative is zero when *x* is at the boundary)

3 $$\begin{aligned} \frac{\partial S_{H}}{\partial \nu }=\frac{\partial I_{H}}{\partial \nu }=\frac{\partial S_{V}}{\partial \nu }=\frac{\partial I_{V}}{\partial \nu }=0,~ x\in \partial \Omega ,~ t>0, \end{aligned}$$

and initial condition

4 $$\begin{aligned} S_{H}(x,0)=S_{H,0}(x), I_{H}(x,0)=I_{H,0}(x), S_{V}(x,0)=S_{V,0}(x), I_{V}(x,0)=I_{V,0}(x), x\in \Omega . \end{aligned}$$

where Eq. (4) represents the value in the individual at the initial time (namely, t=0). $d_{1}$ and $d_{2}$ represent the diffusion coefficients, and $d_{1}>0$, $d_{2}>0$. $\mu _{h}(x)$, $\mu (x)$, $\beta _{H}(x)$, *b*(*x*), $\gamma _{H}(x)$, $\beta _{v}(x)$ and $\nu (x)$ are positive continuous functions on ${\overline{\Omega }}$. The dispersal kernel function ${\mathcal {J}}$ is continuous and satisfies the following properties

5 $$\begin{aligned} {\mathcal {J}}(0)>0,~\int _{R}{\mathcal {J}}(x)dx=1,~{\mathcal {J}} (x)>0~on~{\overline{\Omega }},~{\mathcal {J}}(x)={\mathcal {J}}(-x)\ge 0~on~R. \end{aligned}$$

Let us consider the following function spaces and positive cones.

$$\begin{aligned} \mathbb {X}:=C({\overline{\Omega }}),~\mathbb {X}_{+}:=\mathbb {C}_{+}({\overline{\Omega }}),~\mathbb {Y}:=\mathbb {C}({\overline{\Omega }})\times \mathbb {C}({\overline{\Omega }})\times \mathbb {C}({\overline{\Omega }})\times \mathbb {C}({\overline{\Omega }}),~\mathbb {Y}_{+}:=\mathbb {C}_{+}({\overline{\Omega }})\times \mathbb {C}_{+}({\overline{\Omega }})\times \mathbb {C}_{+}({\overline{\Omega }})\times \mathbb {C}_{+}({\overline{\Omega }}). \end{aligned}$$

$\mathbb {X}$ and $\mathbb {Y}$ are defined as follows, respectively. $\Vert \chi \Vert _{\mathbb {X}}:=\sup _{x\in {\overline{\Omega }}}|\chi (x)|,~\chi \in \mathbb {X},$

$$\begin{aligned} \Vert (g_{1},g_{2},g_{3},g_{4})\Vert _{\mathbb {Y}}:=\sup _{x\in {\overline{\Omega }}}\sqrt{|g_{1}(x)|^{2}+|g_{2}(x)|^{2} +|g_{3}(x))|^{2}+|g_{4}(x))|^{2}},\\ ~(a_{1},a_{2},a_{3},a_{4})\in \mathbb {Y}. \end{aligned}$$

Next, we define the linear operators on $\mathbb {X}$.

6 $$\begin{aligned} \begin{aligned}{}&{\mathcal {A}}_{1}\chi _{1}(x):=d_{1}\int _{\Omega } {\mathcal {J}}(x-y)\chi _{1}(y)dy-d_{1}\chi _{1}(x)-\mu (x)\chi _{1}(x),\\&{\mathcal {A}}_{2}\chi _{2}(x):=d_{2}\int _{\Omega } {\mathcal {J}}(x-y)\chi _{2}(y)dy-d_{2}\chi _{2}(x)-(\mu (x)+\gamma _{H}(x))\chi _{2}(x),\\&{\mathcal {A}}_{3}\chi _{3}(x):=-\nu (x)\chi _{3}(x),\\&{\mathcal {A}}_{4}\chi _{4}(x):=-\nu (x)\chi _{4}(x). \end{aligned} \end{aligned}$$

From the above, we know that ${\mathcal {A}}_{i} (i=1,2,3,4)$ are bounded linear operators, by virtue of^22^, Theorem 1.2, we obtain that ${{\mathcal {A}}_{i}(t)}_{t\ge 0}$ are uniformly continuous semigroups on $\mathbb {X}$. Furthermore, according to^23^, Sect. 2.1.1, the semigroups ${{\mathcal {A}}_{i}(t)}_{t\ge 0}$ are positive.

### Well-posedness of the solution

In this section, we will prove the existence and uniqueness of the solution for system (2).

#### Theorem 2.1

Assuming $(S_{H,0},I_{H,0},S_{V,0}I_{V,0})\in \mathbb {Y}$, system (2) exists the unique solution $(S_{H}(\cdot ,t),I_{H}(\cdot ,t), S_{V}(\cdot ,t)I_{V}(\cdot ,t))$ for all $t\in [0,t_{0})$, and either $t_{0}=+\infty$ or $\limsup _{t\rightarrow t_{0_{-0}}}\Vert (S_{H}(\cdot ,t),I_{H}(\cdot ,t),S_{V}(\cdot ,t),I_{V}(\cdot ,t)\Vert =+\infty$.

#### Proof

$$\begin{aligned} \mathbb {F}(\vartheta _{1},\vartheta _{2},\vartheta _{3},\vartheta _{4})(x) =\left( {\begin{array}{*{10}{c}} \mu _{h}(x)N_{H}-\frac{\beta _{H}(x)b(x)}{N_{H}+m}\vartheta _{1}\vartheta _{4}\\ \frac{\beta _{H}(x)b(x)}{N_{H}+m}\vartheta _{1}\vartheta _{4}\\ A-\frac{\beta _{V}(x)b(x)}{N_{H}+m}\vartheta _{2}\vartheta _{3}\\ \frac{\beta _{V}(x)b(x)}{N_{H}+m}\vartheta _{2}\vartheta _{3}\\ \end{array}}\right) . \end{aligned}$$

Let $\mathbb {F}'[\zeta _{1},\zeta _{2},\zeta _{3},\zeta _{4}]$ be a linear operator on $\mathbb {Y}$ defined as follow:

$$\begin{aligned} \mathbb {F}'[\zeta _{1},\zeta _{2},\zeta _{3},\zeta _{4}](\vartheta _{1}, \vartheta _{2},\vartheta _{3},\vartheta _{4})(x)=\left( {\begin{array}{*{10}{c}} -\frac{\beta _{H}(x)b(x)}{N_{H}+m}\zeta _{4}\vartheta _{1} -\frac{\beta _{H}(x)b(x)}{N_{H}+m}\zeta _{1}\vartheta _{4}\\ \frac{\beta _{H}(x)b(x)}{N_{H}+m}\zeta _{4}\vartheta _{1} +\frac{\beta _{H}(x)b(x)}{N_{H}+m}\zeta _{1}\vartheta _{4}\\ -\frac{\beta _{V}(x)b(x)}{N_{H}+m}\zeta _{3}\vartheta _{2} -\frac{\beta _{V}(x)b(x)}{N_{H}+m}\zeta _{2}\vartheta _{3}\\ \frac{\beta _{V}(x)b(x)}{N_{H}+m}\zeta _{3}\vartheta _{2} +\frac{\beta _{V}(x)b(x)}{N_{H}+m}\zeta _{2}\vartheta _{3}\\ \end{array}}\right) . \end{aligned}$$

By calculating, we have

$$\begin{aligned} \begin{aligned} \mathbb {F}(\vartheta _{1},\vartheta _{2},\vartheta _{3},\vartheta _{4})(x)&=\mathbb {F}(\zeta _{1},\zeta _{2},\zeta _{3},\zeta _{4})(x) +\mathbb {F}'[\zeta _{1},\zeta _{2},\zeta _{3},\zeta _{4}](\vartheta _{1} -\zeta _{1},\vartheta _{2}-\zeta _{2},\vartheta _{3}-\zeta _{3},\vartheta _{4}-\zeta _{4})(x)\\&\quad +\left( {\begin{array}{*{10}{c}} -\frac{\beta _{H}(x)b(x)}{N_{H}+m}(\vartheta _{1}-\zeta _{1})(\vartheta _{4}-\zeta _{4})\\ \frac{\beta _{H}(x)b(x)}{N_{H}+m}(\vartheta _{1}-\zeta _{1})(\vartheta _{4}-\zeta _{4})\\ -\frac{\beta _{V}(x)b(x)}{N_{H}+m}(\vartheta _{2}-\zeta _{2})(\vartheta _{3}-\zeta _{3})\\ \frac{\beta _{V}(x)b(x)}{N_{H}+m}(\vartheta _{2}-\zeta _{2})(\vartheta _{3}-\zeta _{3})\\ \end{array}}\right) , \end{aligned} \end{aligned}$$

due to the coefficients are positive and bounded, we have that the last term in the right-hand of this equation is $o\{(\vartheta _{1},\vartheta _{2},\vartheta _{3}, \vartheta _{4})^{T}-(\zeta _{1},\zeta _{2},\zeta _{3},\zeta _{4})^{T}\}$. It means that $\mathbb {F}$ is Fréchet differentiable for $(\zeta _{1},\zeta _{2},\zeta _{3},\zeta _{4})^{T}$ on $\mathbb {Y}$. Moreover, we have

$$\begin{aligned} \begin{aligned}{}&\Vert \mathbb {F}'[\zeta _{1},\zeta _{2},\zeta _{3},\zeta _{4}] -\mathbb {F}'[{\tilde{\zeta }}_{1},{\tilde{\zeta }}_{2},{\tilde{\zeta }}_{3},{\tilde{\zeta }}_{4}]\Vert \\&\quad =\sup _{\Vert (\vartheta _{1},\vartheta _{2},\vartheta _{3}, \vartheta _{4})^{T}\Vert _{\mathbb {Y}}\le 1}\Vert \{\mathbb {F}' [\zeta _{1},\zeta _{2},\zeta _{3},\zeta _{4}]-\mathbb {F}'[{\tilde{\zeta }}_{1}, {\tilde{\zeta }}_{2},{\tilde{\zeta }}_{3},{\tilde{\zeta }}_{4}]\}(\vartheta _{1}, \vartheta _{2},\vartheta _{3},\vartheta _{4})\Vert _{\mathbb {Y}}\\&\quad =\sup _{\Vert (\vartheta _{1},\vartheta _{2},\vartheta _{3}, \vartheta _{4})^{T}\Vert _{\mathbb {Y}}\le 1}\Vert \{-\frac{\beta _{H}(x)b(x)}{N_{H}+m} (\zeta _{4}-{\tilde{\zeta }}_{4})\vartheta _{1}-\frac{\beta _{H}(x)b(x)}{N_{H}+m} (\zeta _{1}-{\tilde{\zeta }}_{1})\vartheta _{4},\\&\quad \quad \quad \frac{\beta _{H}(x)b(x)}{N_{H}+m}(\zeta _{4}-{\tilde{\zeta }}_{4})\vartheta _{1} +\frac{\beta _{H}(x)b(x)}{N_{H}+m}(\zeta _{1}-{\tilde{\zeta }}_{1})\vartheta _{4}, -\frac{\beta _{V}(x)b(x)}{N_{H}+m}(\zeta _{3}-{\tilde{\zeta }}_{3})\vartheta _{2}\\&\quad \quad -\frac{\beta _{V}(x)b(x)}{N_{H}+m}(\zeta _{2}-{\tilde{\zeta }}_{2})\vartheta _{3},\frac{\beta _{V}(x)b(x)}{N_{H}+m}(\zeta _{3}-{\tilde{\zeta }}_{3})\vartheta _{2} +\frac{\beta _{V}(x)b(x)}{N_{H}+m}(\zeta _{2}-{\tilde{\zeta }}_{2})\vartheta _{3}\}\Vert \\&\quad \le 2\frac{{\bar{\beta }}_{H}{\bar{b}}}{N_{H}+m}\Vert (\zeta _{1}-{\tilde{\zeta }}_{1}, \zeta _{2}-{\tilde{\zeta }}_{2},\zeta _{3}-{\tilde{\zeta }}_{3},\zeta _{4}-{\tilde{\zeta }}_{4})^{T}\Vert _{\mathbb {Y}}, \end{aligned} \end{aligned}$$

where $({\tilde{\zeta }}_{1},{\tilde{\zeta }}_{2},{\tilde{\zeta }}_{3},{\tilde{\zeta }}_{4})^{T}\in \mathbb {Y}$, this implies that $\mathbb {F}$ is continuously Fr$\acute{e}$chet differentiable^24^, Lemma 3.1 on $\mathbb {Y}$.

Due to ${{\mathcal {A}}_{i}(t)}_{t\ge 0}$ are uniformly continuous semigroups, the solution $(S_{H}(x,t),I_{H}(x,t),S_{V}(x,t)I_{V}(x,t))$ of system (2) can be written as follows:

$$\begin{aligned} p(x,t)=e^{{\mathcal {A}}(t)}p(\cdot ,t)(x)+\int _{0}^{t} E^{{\mathcal {A}}(t-s)}\mathbb {F}(w(\cdot ,\tau ))(x)ds,~t\ge 0,~x\in {\bar{\Omega }}, \end{aligned}$$

where

$$\begin{aligned} p(x,t)=\left( {\begin{array}{*{10}{c}} S_{H}(x,t)\\ I_{H}(x,t)\\ S_{V}(x,t)\\ I_{V}(x,t)\\ \end{array}}\right) ,~~ {\mathcal {A}}(t)=\left( {\begin{array}{*{10}{c}} {\mathcal {A}}_{1}(t)\\ {\mathcal {A}}_{2}(t)\\ {\mathcal {A}}_{3}(t)\\ {\mathcal {A}}_{4}(t)\\ \end{array}}\right) ,~~ \mathbb {F}(p(x,t))=\left( {\begin{array}{*{10}{c}} \mu _{h}(x)N_{H}-\frac{\beta _{H}(x)b(x)}{N_{H}+m}S_{H}(x,t)I_{V}(x,t)\\ \frac{\beta _{H}(x)b(x)}{N_{H}+m}S_{H}(x,t)I_{V}(x,t)\\ A-\frac{\beta _{V}(x)b(x)}{N_{H}+m}S_{V}(x,t)I_{H}(x,t)\\ \frac{\beta _{V}(x)b(x)}{N_{H}+m}S_{V}(x,t)I_{H}(x,t)\\ \end{array}}\right) . \end{aligned}$$

Due to ${\mathcal {A}}$ be the infinitesimal generator of ${e^{t{\mathcal {A}}}}_{t\ge 0}$ and $\mathbb {F}$ is continuously Fr$\acute{e}$chet differentiable on $\mathbb {Y}$. From^25^, Proposition 4.16, the result holds. $\square$

#### Lemma 2.1

If $(S_{H}(\cdot ,t),I_{H}(\cdot ,t),S_{V}(\cdot ,t)I_{V}(\cdot ,t))\in \mathbb {Y}$ be the solution of system (2) with $(S_{H,0},I_{H,0},S_{V,0}I_{V,0})\in \mathbb {Y}_{+}$. Then $(S_{H}(\cdot ,t),I_{H}(\cdot ,t),S_{V}(\cdot ,t)I_{V}(\cdot ,t))\in \mathbb {Y}_{+}$ for all $t\in [0,t_{0})$.

#### Proof

By calculation, we have

7 $$\begin{aligned} \begin{aligned} S_{H}(x,t)&=S_{H,0}(x)e^{-\int _{0}^{t}(d_{1}+\mu (x) +\frac{\beta _{H}(x)b(x)}{N_{H}+m}I_{V}(x,u))du}+\int _{0}^{t}\left( d_{1} \int _{\Omega }{\mathcal {J}}(x-y)S_{H}(y,\tau )dy+\mu _{h}(x)N_{H}\right) \\&\quad \times e^{-\int _{\tau }^{t}(d_{1}+\mu (x)+\frac{\beta _{H}(x)b(x)}{N_{H}+m}I_{V}(x,u))du}d\tau ,\\ I_{H}(x,t)&=I_{H,0}(x)e^{-(d_{2}+\mu (x)+\gamma _{H}(x))t} +\int _{0}^{t}\left( d_{2}\int _{\Omega }{\mathcal {J}}(x-y)I_{H}(y,\tau )dy +\frac{\beta _{H}(x)b(x)}{N_{H}+m}S_{H}(x,\tau )I_{V}(x,\tau )\right) \\&\quad \times e^{-(d_{1}+\mu (x)+\gamma _{H}(x))(t-\tau )}d\tau , \end{aligned} \end{aligned}$$

and

8 $$\begin{aligned} \begin{aligned} S_{V}(x,t)&=S_{V,0}(x)e^{-\int _{0}^{t}(\nu (x) +\frac{\beta _{V}(x)b(x)}{N_{H}+m}I_{H}(x,u))du}+A\int _{0}^{t} e^{-\int _{\tau }^{t}(\nu (x)+\frac{\beta _{V}(x)b(x)}{N_{H}+m}I_{H}(x,u))du}d\tau ,\\ I_{V}(x,t)&= I_{V,0}(x)e^{-\nu (x)t}+\int _{0}^{t} \frac{\beta _{V}(x)b(x)}{N_{H}+m}S_{V}(x,\tau )I_{H}(x,\tau ) e^{-\nu (x)(t-\tau )}d\tau . \end{aligned} \end{aligned}$$

For all $t\in [0,t_{0})$ and $x\in {\bar{\Omega }}$. Due to $(S_{H,0},I_{H,0},S_{V,0}I_{V,0})\in \mathbb {Y}_{+}$ and ${\mathcal {J}}(x)\ge 0$ on *R*, it means $S_{H}(x,t)\ge 0$, $I_{H}(x,t)\ge 0$, $S_{V}(x,t)\ge 0$, and $I_{V}(x,t)\ge 0$, further, $S_{H}(\cdot ,t)>0$, $I_{H}(\cdot ,t)>0$, $S_{V}(\cdot ,t)>0$, and $I_{V}(\cdot ,t)>0$ for $t\in [0,t_{0})$. $\square$

#### Lemma 2.2

For any initial data $(S_{H,0},I_{H,0},S_{V,0},I_{V,0})$ and $t\in [0,t_{0})$, the solution $(S_{H}(x,t),I_{H}(x,t), S_{V}(x,t),I_{V}(x,t))$ of system (2) satisfy that

9 $$\begin{aligned} \lim \sup _{t\rightarrow \infty }\int _{\Omega }[S_{H}(x,t)+ I_{H}(x,t)+S_{V}(x,t)+ I_{V}(x,t)]dx<\infty \end{aligned}$$

#### Proof

By (2) and (5), we have

$$\begin{aligned} \begin{aligned}{}&\frac{d}{dt}\int _{\Omega }[S_{H}(x,t)+ I_{H}(x,t)+S_{V}(x,t)+ I_{V}(x,t)]dx\\&\quad =d_{1}\int _{\Omega }\int _{\Omega }{\mathcal {J}}(x-y)S_{H}(y,t)dydx-d_{1} \int _{\Omega }S_{H}(x,t)dx+\int _{\Omega }\mu _{h}(x)N_{H}dx-\int _{\Omega }\mu (x) S_{H}(x,t)dx\\&\quad \quad -\int _{\Omega }\frac{\beta _{H}(x)b(x)}{N_{H}+m}S_{H}(x,t)I_{V}(x,t)dx +d_{2}\int _{\Omega }\int _{\Omega }{\mathcal {J}}(x-y)I_{H}(y,t)dydx-d_{2}\int _{\Omega }I_{H}(x,t)dx\\&\quad \quad + \int _{\Omega }\frac{\beta _{H}(x)b(x)}{N_{H}+m}S_{H}(x,t)I_{V}(x,t)dx -\int _{\Omega }(\mu (x)+\gamma _{H}(x))I_{H}(x,t)dx\int _{\Omega }A(x)dx-\int _{\Omega }\nu (x) S_{V}(x,t)dx\\&\quad \quad -\int _{\Omega }\frac{\beta _{V}(x)b(x)}{N_{H}+m}S_{V}(x,t)I_{H}(x,t)dx +\int _{\Omega }\frac{\beta _{V}(x)b(x)}{N_{H}+m}S_{V}(x,t)I_{H}(x,t)dx-\int _{\Omega }\nu (x) I_{V}(x,t)dx\\&\quad = d_{1}\int _{\Omega }\int _{\Omega }{\mathcal {J}}(x-y)S_{H}(y,t)dydx -d_{1}\int _{\Omega }S_{H}(x,t)dx+\int _{\Omega }\mu _{h}(x)N_{H}dx -\int _{\Omega }\mu (x) S_{H}(x,t)dx\\&\quad \quad +d_{2}\int _{\Omega }\int _{\Omega }{\mathcal {J}}(x-y)I_{H}(y,t)dydx -d_{2}\int _{\Omega }I_{H}(x,t)dx-\int _{\Omega }(\mu (x)+\gamma _{H}(x))I_{H}(x,t)dx\\&\quad \quad +\int _{\Omega }A(x)dx-\int _{\Omega }\nu (x) S_{V}(x,t)dx-\int _{\Omega }\nu (x) I_{V}(x,t)dx. \end{aligned} \end{aligned}$$

Furthermore, we have

$$\begin{aligned} \begin{aligned}{}&\frac{d}{dt}\int _{\Omega }[S_{H}(x,t)+ I_{H}(x,t)+S_{V}(x,t)+ I_{V}(x,t)]dx\\&\quad \le d_{1}\int _{\Omega }\int _{\Omega }{\mathcal {J}}(x-y)dyS_{H}(y,t)dx-d_{1} \int _{\Omega }S_{H}(x,t)dx+\int _{\Omega }\mu _{h}(x)N_{H}dx-\int _{\Omega }\mu (x) S_{H}(x,t)dx\\&\quad \quad +d_{2}\int _{\Omega }\int _{\Omega }{\mathcal {J}}(x-y)dyI_{H}(y,t)dx-d_{2} \int _{\Omega }I_{H}(x,t)dx-\int _{\Omega }(\mu (x)+\gamma _{H}(x))I_{H}(x,t)dx\\&\quad \quad +\int _{\Omega }A(x)dx-\int _{\Omega }\nu (x) S_{V}(x,t)dx-\int _{\Omega }\nu (x) I_{V}(x,t)dx\\&\quad \le ({\bar{\mu }}_{h}N_{H}+{\bar{A}})|\Omega |-\int _{\Omega }min\{{\underline{\mu }},{\underline{\nu }}\}( S_{H}(x,t)+I_{H}(x,t)+S_{V}(x,t)+ I_{V}(x,t))dx, \end{aligned} \end{aligned}$$

where $|\Omega |$ denotes the volume of $\Omega$. By virtue of the variation of constants formula and take limit as $t\rightarrow \infty$, we can obtain that

$$\begin{aligned} \lim \sup _{t\rightarrow \infty }\int _{\Omega }[S_{H}(x,t)+ I_{H}(x,t)+S_{V}(x,t)+ I_{V}(x,t)]dx\le \frac{({\bar{\mu }}_{h}N_{H}+{\bar{A}})|\Omega |}{min\{{\underline{\mu }},{\underline{\nu }}\}}. \end{aligned}$$

$$\square $$

### Basic reproduction number

For a more abstract representation of the basic reproduction number, we utilize the next-generation matrix method^26^ and evaluate the linearized equations surrounding the disease-free equilibrium $E^{0}=({S_{H}^{0}(x),0,S_{V}^{0}(x),0})$:

10 $$\begin{aligned} {\left\{ \begin{array}{ll} \frac{\partial I_{H}}{\partial t}=d_{2}\int _{\Omega }{\mathcal {J}} (x-y)I_{H}(y,t)dy-d_{2}I_{H}(x,t)+\frac{\beta _{H}(x)b(x)}{ N_{H}+m}S_{H}^{0}(x)I_{V}(x,t)-(\mu (x)+\gamma _{H}(x))I_{H}(x,t), \\ \frac{\partial I_{V}}{\partial t}=\frac{\beta _{V}(x)b(x)}{N_{H}+m} S_{V}^{0}(x)I_{H}(x,t)-\nu (x) I_{V}(x,t),\\ x\in {\bar{\Omega }},~~t>0. \\ \end{array}\right. } \end{aligned}$$

System (10) be equivalent to

$$\begin{aligned} \frac{\partial \eta }{\partial t}=B\eta -D\eta +G\eta ,~~x\in \Omega ,t>0, \end{aligned}$$

where

$$\begin{aligned} \eta =\left( {\begin{array}{*{10}{l}} I_{H}\\ I_{V}\\ \end{array}}\right) ,~~ B=\left( {\begin{array}{*{10}{c}} &{}d_{2}\int _{\Omega }{\mathcal {J}}(x-y)dy~~~~~&{}0\\ &{}0~~~~~&{}0\\ \end{array}}\right) , \end{aligned}$$

  and  

$$\begin{aligned} D=\left( {\begin{array}{*{10}{c}} \gamma _{H}+\mu +d_{2}~~~~0\\ 0~~~~~~~~~~~~~~~~~~~~\nu \\ \end{array}}\right) ~~ G=\left( {\begin{array}{*{10}{c}} 0~~~~~~~~~~~~\frac{\beta _{H}b}{N_{H}+m}S^{0}_{H}\\ \frac{\beta _{V}b}{N_{H}+m}S^{0}_{V}~~~~~~~~~~~~0\\ \end{array}}\right) . \end{aligned}$$

By virtue of^27^, Chapter 11, we obtain that the following linear equation

11 $$\begin{aligned} \frac{\partial \eta }{\partial t}=(B-D)\eta ,~~x\in \Omega ,t>0. \end{aligned}$$

Let *T*(*t*) be the solution semigroup with respect to the linear Eq. (11). Define

$$\begin{aligned} \mathbb {K}(\vartheta )(x):=\int _{0}^{\infty }G[T(t)(\vartheta )](x)dt. \end{aligned}$$

In terms of the next infection operator, the spectral radius of $\mathbb {K}$ can be defined as the basic reproduction number

$$\begin{aligned} R_{0}:=r(\mathbb {K}). \end{aligned}$$

We consider the following eigenvalue problem with respect to system (10).

12 $$\begin{aligned} {\left\{ \begin{array}{ll} \lambda \Phi (x)=d_{2}\int _{\Omega }{\mathcal {J}}(x-y) \Phi (y)dy-d_{2}\Phi (x)+\frac{\beta _{H}(x)b(x)}{N_{H}+m} S_{H}^{0}(x)\Psi (x)-(\mu (x)+\gamma _{H}(x))\Phi (x), \\ \lambda \Psi (x)=\frac{\beta _{V}(x)b(x)}{N_{H}+m}S_{V}^{0} (x)\Phi (x)-\nu (x) \Psi (x)).\\ \end{array}\right. } \end{aligned}$$

Meanwhile, by virtue of^28^, for system (12), there exists a principal eigenvalue $\lambda _{0}$ with respect to a pair positive continuous eigenfunction $(\Phi _{0}(x),\Psi _{0}(x))$ satisfy that the following lemma.

#### Lemma 2.3

$$sign(R_{0}-1 ) = sign \lambda _{0}$$.

#### Proof

The proof procedure can be referred to reference^14^, Theorem 2.10. $\square$

## Global stability and uniform persistence

### Global stability of the disease-free equilibrium

Global stability of the disease-free equilibrium is to be demonstrated. Before proving its global asymptotic stability, certain lemmas are presented. Additionally, we investigate an eigenvalue problem previously examined Garc$\acute{i}$a-Meli$\acute{a}$n and Rossi^13^.

13 $$\left\{ {\begin{array}{*{20}l}    {\int _{R^{N}}{\mathcal {J}}(x-y)(\varrho (y)-\varrho (x))dy =-\lambda _{e}\varrho (x),} \hfill & {in~\;\Omega ,} \hfill  \\    {\varrho (x)=0,} \hfill & {on\;~R^{N} { \setminus }\Omega ,} \hfill  \\   \end{array} } \right. $$

#### Lemma 3.1

For system (13), there exists a unique principal eigenvalue $\lambda _{1}$ correspond to eigenfunction $\varrho (x)$. Furthermore, $0<\lambda _{1}<1$ and

$$\begin{aligned} \lambda _{1}=\inf _{\varrho \in L^{2}(\Omega ),\varrho \ne 0}\frac{\int _{\Omega }\varrho ^{2}(x)dx -\int _{\Omega }\int _{\Omega }{\mathcal {J}}(x-y)\varrho (y)\varrho (x)dydx}{\int _{\Omega }\varrho ^{2}(x)}. \end{aligned}$$

Now, we have the following global stability result.

#### Theorem 3.1

If $R_{0}<1$, the solution $(S_{H}(x,t),I_{H}(x,t),S_{V}(x,t)I_{V}(x,t))$ of system (2) converge to the disease-free equilibrium $({S_{H}^{0}(x),0,S_{V}^{0}(x),0})$ on *x* as $t\rightarrow +\infty$.

#### Proof

We first prove that $S_{H}(x,t)\rightarrow S_{H}^{0}(x)$ on *x* as $t\rightarrow +\infty$, let $h_{1}(x,t)=S_{H}(x,t)-S_{H}^{0}(x)$. Furthermore, we have

14 $$\begin{aligned} \frac{\partial h_{1}(x,t)}{\partial t}=d_{1}\int _{\Omega }{\mathcal {J}}(x-y)h_{1}(y,t)dy-d_{1}h_{1}(x,t)-\mu (x) h_{1}(x,t)-\frac{\beta _{H}(x)b(x)}{N_{H}+m}S_{H}(x,t)I_{V}(x,t),~x\in \Omega . \end{aligned}$$

Let $H(t)=\int _{\Omega }h_{1}^{2}(x,t)dx$, we can obtain

15 $$\begin{aligned} \begin{aligned}{}&\frac{dH(t)}{dt}\\&\quad =2\int _{\Omega }h_{1}(x,t)\frac{\partial h_{1}(x,t)}{\partial t}dx\\&\quad = 2\int _{\Omega }h_{1}(x,t)\left\{ d_{1}\int _{\Omega }{\mathcal {J}}(x-y) h_{1}(y,t)dy-d_{1}h_{1}(x,t)-\mu (x) h_{1}(x,t)-\frac{\beta _{H}(x) b(x)}{N_{H}+m}S_{H}(x,t)I_{V}(x,t)\right\} dx\\&\quad = 2\left\{ d_{1}\int _{\Omega }\int _{\Omega }{\mathcal {J}}(x-y)h_{1}(y,t) h_{1}(x,t)dydx-\int _{\Omega }h_{1}^{2}(x,t)dx\right\} \\&\quad \quad -2\int _{\Omega }\left\{ \mu (x) h_{1}(x,t)+\frac{\beta _{H}(x)b(x)}{N_{H}+m}S_{H}(x,t)I_{V}(x,t)\right\} h_{1}(x,t)dx\\&\quad \le -2d_{1}\lambda _{1}H(t). \end{aligned} \end{aligned}$$

By calculation yields that

$$\begin{aligned} H(t)\le c_{0}e^{-2d_{1}\lambda _{1}t}. \end{aligned}$$

Hence, there exists constant $c_{0}$, we have

$$\begin{aligned} \Vert h_{1}(\cdot ,t)\Vert _{L^{2}(\Omega )}\le c_{0}e^{-d_{1}\lambda _{1}t}. \end{aligned}$$

By virtue of Eq. (14), we can obtain

16 $$\begin{aligned} h_{1}(x,t)=h_{0}(x)e^{-(d_{1}+\mu (x))t}+e^{-(d_{1} +\mu (x))t}\int _{0}^{t}e^{d_{1}s}\left( d_{1}\int _{\Omega }{\mathcal {J}} (x-y)h_{1}(y,s)dy-\frac{\beta _{H}(x)b(x)}{N_{H}+m}S_{H}(x,t)I_{V}(x,t)\right) ds. \end{aligned}$$

Applying the h$\ddot{o}$lder inequality to the following equation, there exists some positive constant satisfy that

17 $$\begin{aligned} \int _{\Omega }J(x-y)h_{1}(y,s)dy\le C\Vert h_{1}(\cdot ,s)\Vert _{L^{2}(\Omega )}. \end{aligned}$$

Combine (16) and (17), there exists some positive constants $c_{i}(i=1,2)$ we have

$$\begin{aligned} |h_{1}(x,t)|\le c_{1}e^{-(d_{1}+\mu (x))t}+c_{2}e^{-(d_{1}+\mu (x))\lambda _{1}t}. \end{aligned}$$

Hence, as $t\rightarrow \infty$, $h_{1}(x,t)\rightarrow 0$ uniformly on $x\in \Omega$. Furthermore, we obtain that $S_{H}(x,t)\rightarrow S_{H}^{0}(x)$.

Next, we prove $I_{H}(x,t)\rightarrow 0$, let $V_{1}(t):=\int _{\Omega }I^{2}_{H}(x,t)dx$, we obtain that

18 $$\begin{aligned} \begin{aligned} \frac{dV_{1}(t)}{dt}&=\int _{\Omega }2I_{H}(x,t)\frac{\partial }{\partial t}I_{H}(x,t)dx\\&=\int _{\Omega }2I_{H}(x,t)\left\{ d_{2}\int _{\Omega }{\mathcal {J}} (x-y)I_{H}(y,t)dy-d_{2}I_{H}(x,t)+\frac{\beta _{H}(x)b(x)}{N_{H}+m}S_{H}(x,t)I_{V}(x,t) \right. \\&\quad -\left. (\mu (x)+\gamma _{H}(x))I_{H}(x,t)\right\} dx\\&=2d_{2}\left\{ \int _{\Omega }\int _{\Omega }{\mathcal {J}}(x-y)I_{H}(y,t) I_{H}(x,t)dydx-\int _{\Omega }I^{2}_{H}(x,t)dx \right\} +2\int _{\Omega } \left\{ \frac{\beta _{H}(x)b(x)}{N_{H}+m}S_{H}(x,t)I_{V}(x,t) \right. \\&\quad -(\mu (x)+\gamma _{H}(x))I_{H}(x,t)\}I_{H}(x,t)dx\\ \end{aligned} \end{aligned}$$

Due to $\beta _{H},b,\mu \in C({\bar{\Omega }})$, by virtue of the above argument, there exists some positive constant $c_{0}>0$ satisfy that

$$\begin{aligned} \left| \frac{\beta _{H}(x)b(x)}{N_{H}+m}S_{H}(x,t)I_{V}(x,t)-(\mu (x)+\gamma _{H}(x))I_{H}(x,t) \right| \le c_{0}e^{\lambda _{0}t}. \end{aligned}$$

Hence, equation (18) be equivalent to

$$\begin{aligned} \frac{dV_{1}(t)}{dt}\le -2d_{2}\lambda _{1}V_{1}(t)+2c_{0}|\Omega |^{\frac{1}{2}}e^{\lambda _{0}t}V_{1}^{\frac{1}{2}}(t). \end{aligned}$$

By calculation yields that

$$\begin{aligned} V_{1}(t)\le \left\{ \begin{aligned}{}&\left( V_{1}^{\frac{1}{2}}(0)+c_{0}|\Omega |^{\frac{1}{2}}t\right) ^{2}e^{-2d_{2}\lambda _{1}t}, ~ if~\lambda _{0}+d_{2}\lambda _{1}=0,\\&\left( \frac{c_{0}|\Omega |^{\frac{1}{2}}}{\lambda _{0}+d_{2}\lambda _{1}}e^{\lambda _{0}t}+\left( V_{1}^{\frac{1}{2}}(0)+\frac{c_{0}|\Omega |^{\frac{1}{2}}}{\lambda _{0}+d_{1}\lambda _{1}}\right) e^{-d_{1}\lambda _{1}t}\right) ^{2}, ~ if~ \lambda _{0}+d_{2}\lambda _{1}\ne 0.\\ \end{aligned} \right. \end{aligned}$$

Hence, for some positive constants $k_{i}(i=1,2,3,4)$, we have

$$\begin{aligned} \Vert I_{H}(\cdot ,t)\Vert _{L^{2}(\Omega )}\le \left\{ \begin{aligned}{}&(k_{1}+k_{2}t)e^{-d_{2}\lambda _{1}t}, ~ if~\lambda _{0}+d_{2}\lambda _{1}=0,\\&k_{3}e^{\lambda _{0}t}+k_{4}e^{-d_{2}\lambda _{1}t}, ~ if~ \lambda _{0}+d_{2}\lambda _{1}\ne 0.\\ \end{aligned} \right. \end{aligned}$$

By virtue of system (2), we can obtain

19 $$\begin{aligned} \begin{aligned} I_{H}(x,t)&=I_{H,0}(x)e^{-d_{2}t}+e^{-d_{2}t}\int _{0}^{t}e^{d_{2}s}(d_{2}\int _{\Omega }{\mathcal {J}}(x-y)I_{H}(y,s)dy\\&\quad +\frac{\beta _{H}(x)b(x)}{N_{H}+m}S_{H}(x,t)I_{V}(x,t)-(\mu (x)+\gamma _{H}(x))I_{H}(x,t))ds. \end{aligned} \end{aligned}$$

Applying the h$\ddot{o}$lder inequality to the following equation, there exists some positive constant satisfy that

20 $$\begin{aligned} \int _{\Omega }{\mathcal {J}}(x-y)I_{H}(y,s)dy\le C\Vert I_{H}(\cdot ,s)\Vert _{L^{2}(\Omega )}. \end{aligned}$$

Combine (19) and (20), there exists some positive constants ${\tilde{k}}_{i}(i=1,2,3,4)$ we have

$$\begin{aligned} |I_{H}(x,t)|\le \left\{ \begin{aligned}{}&{\tilde{k}}_{1}e^{-d_{2}t}+({\tilde{k}}_{2}+{\tilde{k}}_{3}t)e^{-d_{2}\lambda _{1}t}+{\tilde{k}}_{4}e^{\lambda _{0}t}, ~ if~\lambda _{0}+d_{2}\lambda _{1}=0,\\&{\tilde{k}}_{5}e^{-d_{2}t}+{\tilde{k}}_{6}e^{\lambda _{0}t}+{\tilde{k}}_{7}e^{-d_{2}\lambda _{1}t}, ~ if~ \lambda _{0}+d_{2}\lambda _{1}\ne 0.\\ \end{aligned} \right. \end{aligned}$$

Since $R_{0}<1$, we know that $\lambda _{0}<0$, hence, as $t\rightarrow \infty$, $I_{H}(x,t)\rightarrow 0$ uniformly on $x\in \Omega$.

Moreover, we prove that $S_{V}(x,t)\rightarrow S_{V}^{0}(x)$ on *x* as $t\rightarrow +\infty$, let $h_{2}(x,t)=S_{V}(x,t)-S_{V}^{0}(x)$, then, we have

21 $$\begin{aligned} \frac{\partial h_{2}(x,t)}{\partial t}=-\nu (x) h_{2}(x,t)-\frac{\beta _{V}(x)b(x)}{N_{H}+m}S_{V}(x,t)I_{H}(x,t),~x\in \Omega . \end{aligned}$$

Due to $I_{H}(x,t)\rightarrow 0$ as $t\rightarrow \infty$, by virtue of the above argument, we know that $h_{2}(x,t)\rightarrow 0$ as $t\rightarrow \infty$. using the the constant variation method with respect to the last equation of (2), we can obtain that $I_{V}(x,t)\rightarrow 0$ as $t\rightarrow \infty$. $\square$

### Uniform persistence

In this section, we consider the uniform persistence of system (2). To get these goals, we first consider the following problem.

#### Theorem 3.2

For $R_{0}>1$, then there exists a function $\Gamma (x)$, such that

$$\begin{aligned} \begin{aligned}{}&\lim _{t\rightarrow \infty }infS_{H}(x,t)\ge \Gamma (x), \lim _{t\rightarrow \infty }infI_{H}(x,t)\ge \Gamma (x),\\&\lim _{t\rightarrow \infty }infS_{V}(x,t)\ge \Gamma (x), \lim _{t\rightarrow \infty } infI_{V}(x,t)\ge \Gamma (x), \end{aligned} \end{aligned}$$

hence, the disease uniform persistence.

#### Proof

Due to $R_{0}>1$, then, there exists a $\kappa >0$ such that $\lambda (S^{*}_{H}-\kappa ,S^{*}_{V}-\kappa )>0$ (where $(S^{*}_{H},I^{*}_{H},S^{*}_{V},I^{*}_{V})$ represents that the endemic equilibrium ). It means that there exists a ${\widetilde{t}}_{1}>0$ satisfy that $S_{H}(x,t)>S_{H,0}-\kappa$ and $S_{V}(x,t)>S_{V,0}-\kappa$ for $t\ge {\widetilde{t}}_{1}$ and $x\in {\overline{\Omega }}$. For $x\in \Omega ,~~t>{\widetilde{t}}_{1}$, according to the comparison principle, we can obtain

$$\begin{aligned} {\left\{ \begin{array}{ll} \frac{\partial I_{H}}{\partial t}\ge d_{2}\int _{\Omega }{\mathcal {J}} (x-y)I_{H}(y,t)dy-d_{2}I_{H}(x,t)+\frac{\beta _{H}b}{N_{H}+m}(S_{H,0}-\kappa )I_{V}(x,t)-(kN_{H}+\mu +\gamma _{H})I_{H}(x,t), \\ \frac{\partial I_{V}}{\partial t}\ge \frac{\beta _{V}b}{N_{H}+m} (S_{V,0}-\kappa )(x,t)I_{H}(x,t)-\nu I_{V}(x,t). \\ \end{array}\right. } \end{aligned}$$

Define $({\widetilde{I}}_{H}(x,t),{\widetilde{I}}_{V}(x,t),)=(Me^{{\widetilde{\lambda }}t}{\widetilde{\varrho }}_{1}(x),Me^{{\widetilde{\lambda }}t}{\widetilde{\varrho }}_{2}(x))$, $({\widetilde{I}}_{H}(x,t),{\widetilde{I}}_{V}(x,t),)$ satisfy that the following equation

$$\begin{aligned} {\left\{ \begin{array}{ll} \frac{\partial {\widetilde{I}}_{H}}{\partial t}=d_{2}\int _{\Omega } {\mathcal {J}}(x-y){\widetilde{I}}_{H}(y,t)dy-d_{2}{\widetilde{I}}_{H}(x,t) +\frac{\beta _{H}b}{N_{H}+m}(S_{H,0}-\kappa ){\widetilde{I}}_{V}(x,t)-(kN_{H}+\mu +\gamma _{H}){\widetilde{I}}_{H}(x,t), \\ \frac{\partial {\widetilde{I}}_{V}}{\partial t}= \frac{\beta _{V}b}{N_{H}+m} (S_{V,0}-\kappa )(x,t){\widetilde{I}}_{H}(x,t)-\nu {\widetilde{I}}_{V}(x,t), \\ \end{array}\right. } \end{aligned}$$

where $({\widetilde{\varrho }}_{1}(x),{\widetilde{\varrho }}_{2}(x))$ is the eigenfunction with respect to ${\widetilde{\lambda }}<0$. According to the comparison principle, we know $I_{H}(x,t)\ge {\widetilde{I}}_{H}(x,t)$, $I_{V}(x,t)\ge {\widetilde{I}}_{V}(x,t)$ for $x\in \Omega ,t>{\widetilde{t}}_{1}$. Therefore, $I_{H}(x,t)\ge Me^{{\widetilde{\lambda }}t}{\widetilde{\varrho }}_{1}(x)$, $I_{V}(x,t)\ge Me^{{\widetilde{\lambda }}t}{\widetilde{\varrho }}_{2}(x)$ such that

$$\begin{aligned} \lim _{t\rightarrow \infty }infI_{H}(x,t)\ge M{\widetilde{\varrho }}_{1}(x),~ \lim _{t\rightarrow \infty }infI_{V}(x,t)\ge M{\widetilde{\varrho }}_{2}(x). \end{aligned}$$

On the basis of the Lemma (2.2), we know that there exists a constants $K>0$ and ${\widetilde{t}}_{2}$ such that

$$\begin{aligned} I_{V}(x,t)\le K,~I_{H}(x,t)\le K,~t\ge {\widetilde{t}}_{2},~x\in {\overline{\Omega }}. \end{aligned}$$

Then, $S_{H}$ and $S_{V}$ satisfy that the following equation

$$\begin{aligned} {\left\{ \begin{array}{ll} \frac{\partial S_{H}}{\partial t}\ge d_{1}\int _{\Omega }{\mathcal {J}}(x-y)S_{H}(y,t)dy-d_{1}S_{H}(x,t)+\mu _{h}N_{H}-\left( \mu kN_{H}+\frac{\beta _{H}bK}{N_{H}+m}\right) S_{H}(x,t),~~~~~~x\in \Omega ,t>{\widetilde{t}}_{2},\\ \frac{\partial S_{V}}{\partial t}\ge A-\left( \nu +\frac{\beta _{V}bK}{N_{H}+m}\right) S_{V}(x,t),~~~~~~~~~~~~~~~~~~~x\in \Omega ,t>{\widetilde{t}}_{2}.\\ \end{array}\right. } \end{aligned}$$

Hence

$$\begin{aligned} \begin{aligned}{}&\lim _{t\rightarrow \infty }infS_{H}(x,t)\ge (d_{1}K+\mu _{h}N_{H})/\left( d_{1}+\mu kN_{H}+\frac{\beta _{H}bK}{N_{H}+m}\right) ,\\&\lim _{t\rightarrow \infty }infS_{V}(x,t)\ge A/\left( \nu +\frac{\beta _{V}bK}{N_{H}+m}\right) . \end{aligned} \end{aligned}$$

Let $\Gamma (x):=min\{(d_{1}K+\mu _{h}N_{H})/(d_{1}+\mu kN_{H}+\frac{\beta _{H}bK}{N_{H}+m}),~A/(\nu +\frac{\beta _{V}bK}{N_{H}+m}),~M{\widetilde{\varrho }}_{1}(x),~M{\widetilde{\varrho }}_{2}(x)\}$. The disease uniform persistence is obtained. $\square$

## Numerical simulations

This section presents the theoretical results supported by numerical simulations are presented in this section. The parameter values and initial value are chosen as follows:

**Table 2:** The parameter values.

Parameter	Value	Parameter	Value
*b*	0.76*30.4 $(\textrm{Month}^{-1})$^8^	$N_{H}+m$	100^11^
$\mu$	0.001574^2,9^	*A*	2.5
$\nu$	30.4/14.49 $(\textrm{Month}^{-1})$^8^	$\mu _{h}N_{H}$	6.5
$\gamma _{H}$	1.4 $(\textrm{Month}^{-1})$^10^	$d_{1}$	0.015 $(\textrm{km}^{2}\textrm{Month}^{-1})$^10,11^
$$d_{2} $$	0.015 $$ (\textrm{km}^{2}\textrm{Month}^{-1})$$^10,11^	$$\beta _{V}$$	0.75 $$(1-0.65sinx)$$

initial value:

$$\begin{aligned} (S_{H,0}(x),I_{H,0}(x),S_{V,0}(x),I_{V,0}(x))= \left( 5+cos\frac{\pi x}{2},1.01+sin\frac{\pi x}{2},2+cos\frac{\pi x}{2},0.95+sin\frac{x}{2} \right) . \end{aligned}$$

Moreover, the nonlocal kernel function^23^ is selected as follows:

$$\begin{aligned} {\mathcal {J}}(x)= \left\{ \begin{aligned}{}&B exp\left( \frac{1}{x^{2}-1}\right) , ~ -1<x<1,\\&0, ~ otherwise.\\ \end{aligned} \right. \end{aligned}$$

**Figure 1:**
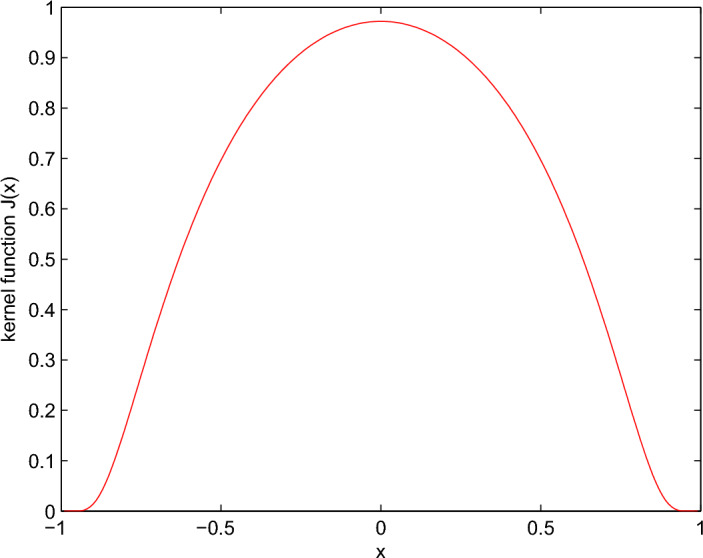
The evolution path of kernel function *J*(*x*).

Here, $B=2.6423$, $x\in [-1,1]\subset R$ and $\int _{R}{\mathcal {J}}(x)dx=\int _{-1}^{1}{\mathcal {J}}(x)dx\approx 1.$ See Fig. 1 for the evolution path of kernel function *J*(*x*).

### Global dynamics of system (2)

**Figure 2:**
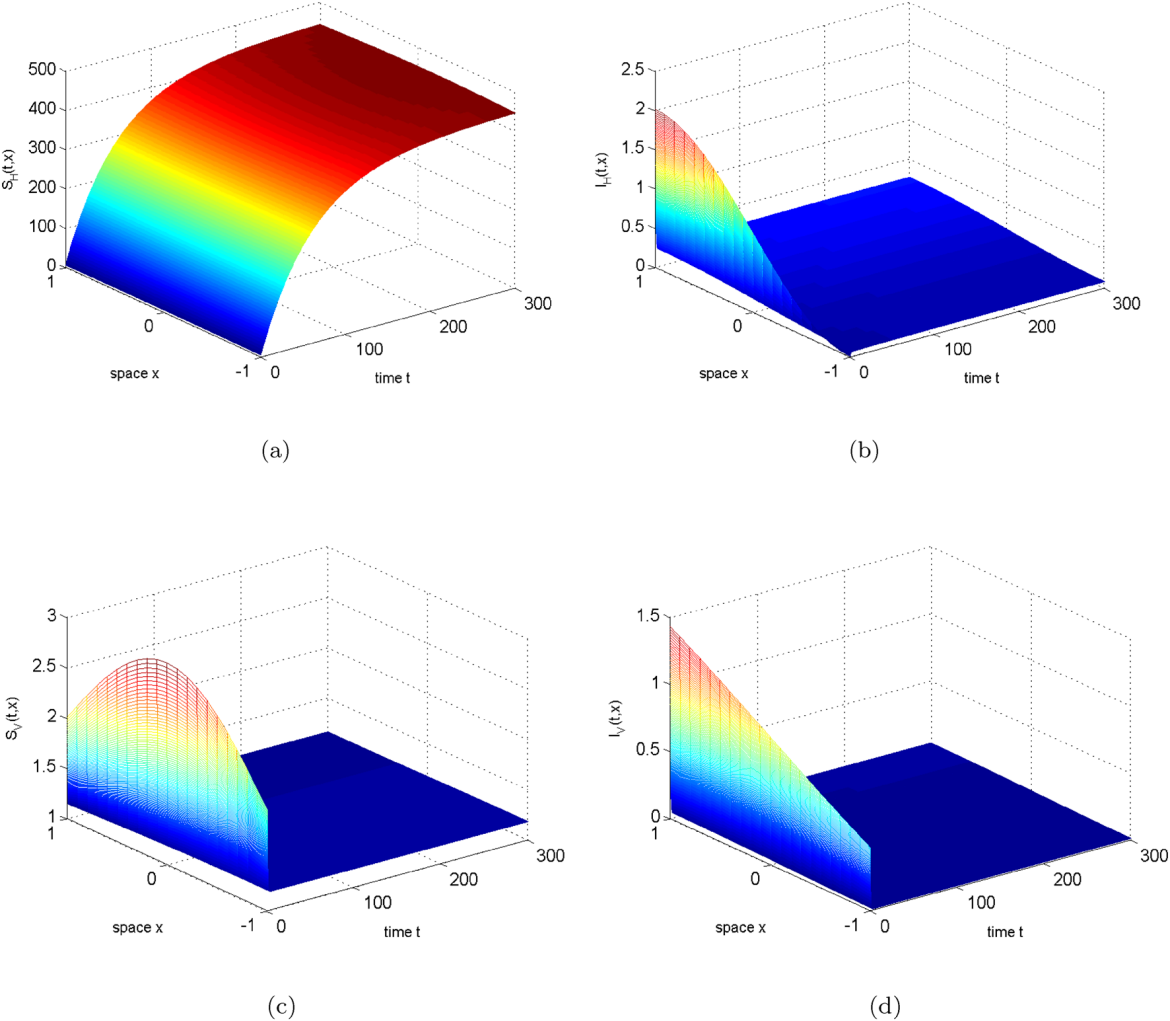
The evolution path of $S_{H}, I_{H}, S_{V}, I_{V}$ for system (2) with $R_0=0.949319338848686<1$.

**Figure 3:**
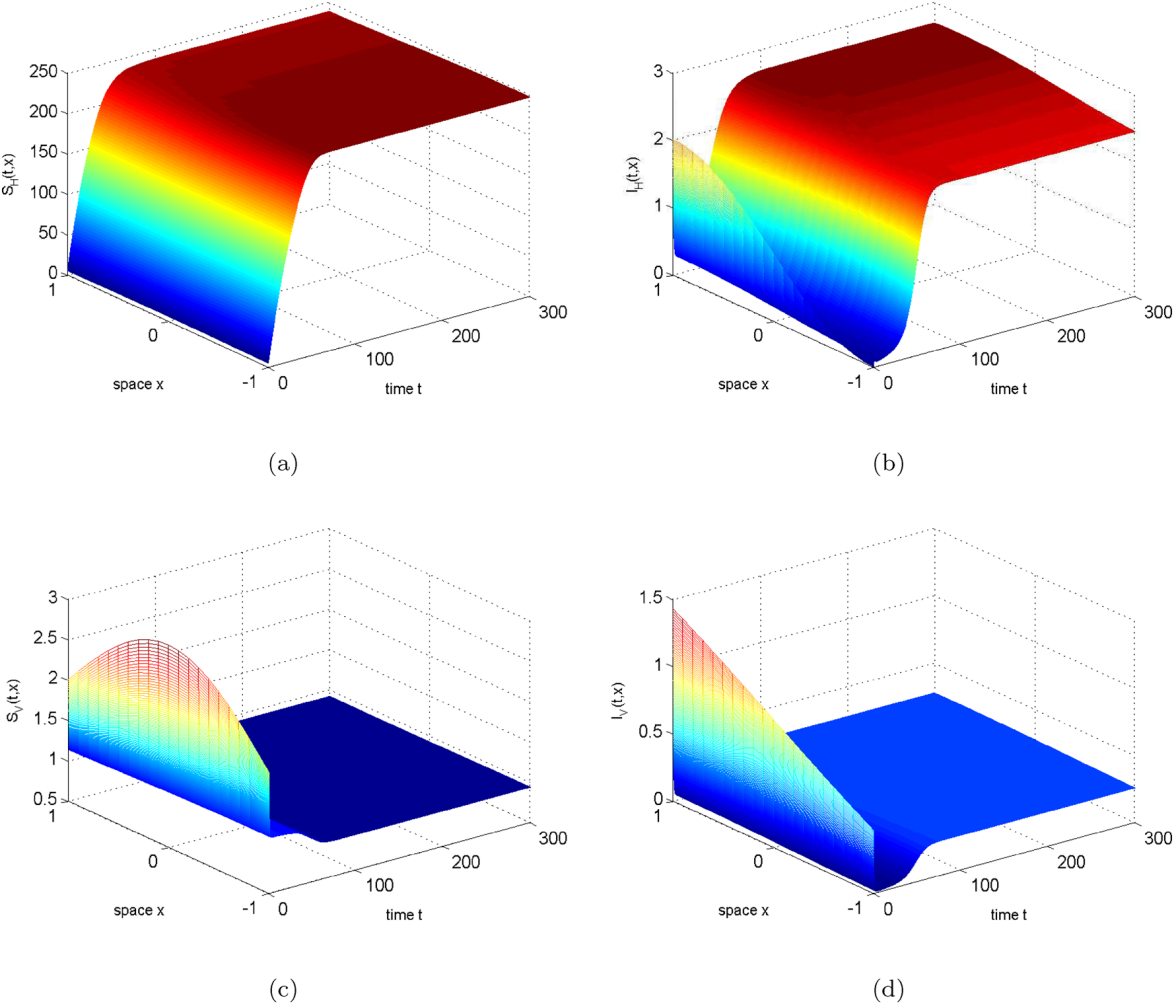
The evolution path of $S_{H}, I_{H}, S_{V}, I_{V}$ for system (2) with $R_0=3.002011337607015>1$.

In this section, we choose to change $\beta _{H}$ to illustrate the result of the theorem. Let $\beta _{H}=0.015(1-0.65cosx)$ and see Table 2 for other parameters, then $R_{0}=0.949319338848686<1$. Figure 2 illustrates the long-term dynamic behavior of the system (2). As time *t* approaches infinity, the density of infected humans and mosquitoes both converge to 0, indicating the extinction of the disease. If the human transmission rate $\beta _{H}$ increases to $10\beta _{H}$, we can obtain $R_0=3.002011337607015>1$. At this point, Fig. 3 shows that the solution of system (2) eventually stabilizes, implying disease persistence.

### The impacts of diffusion rate for infected humans and infected mosquitoes

**Figure 4:**
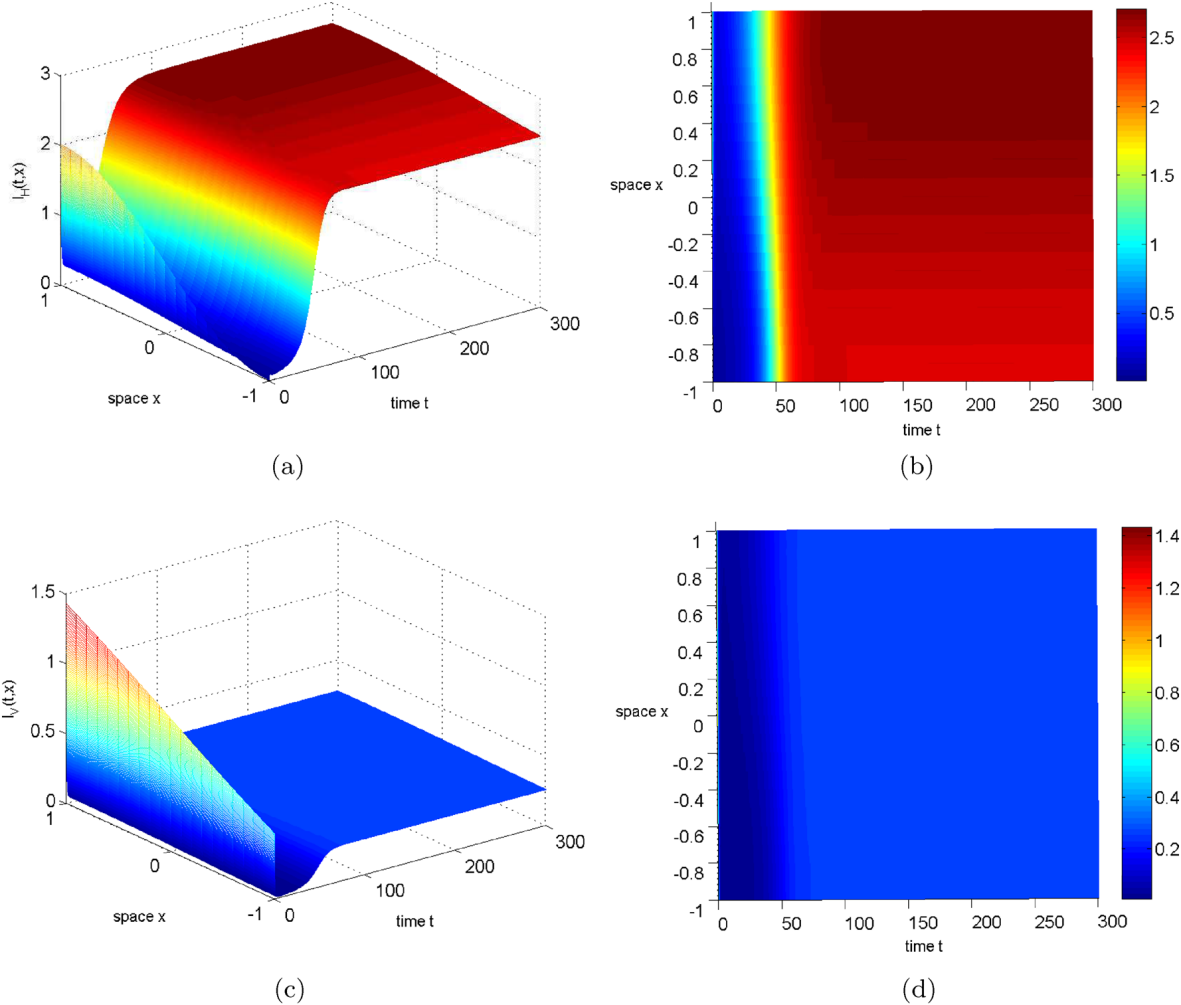
Numerical simulation of $I_{H},I_{V}$ for system (2) with $d_1=d_2=0.015$ (where $R_0=3.002011337607015>1$). Left: The evolution path of $I_{H},I_{V}$. Right: The distribution of $I_{H},I_{V}$ in time and space.

**Figure 5:**
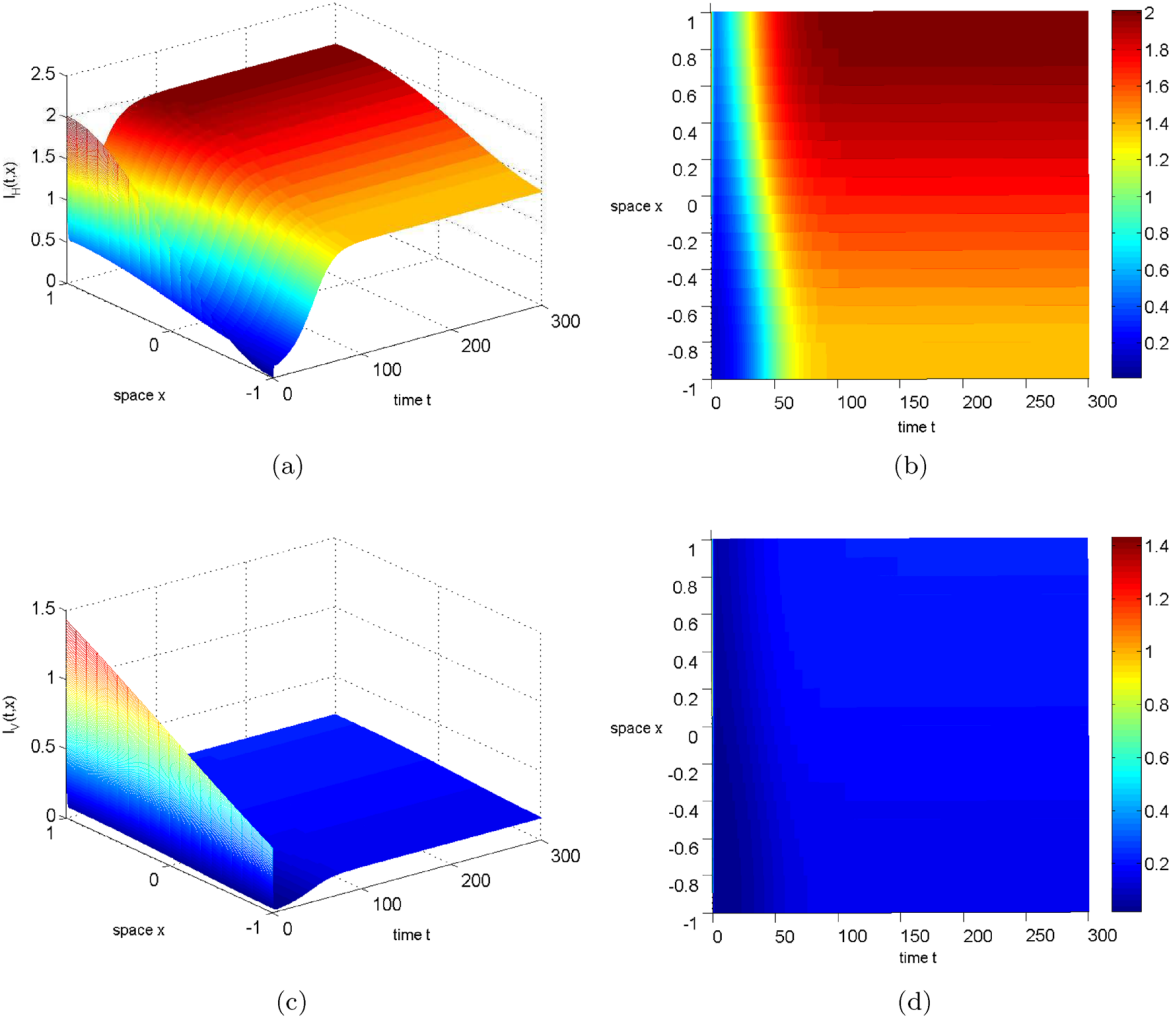
Numerical simulation of $I_{H},I_{V}$ for system (2) with $d_1=d_2=0.030$ (where $R_0=2.986675318735982>1$). Left: The evolution path of $I_{H},I_{V}$. Right: The distribution of $I_{H},I_{V}$ in time and space.

**Figure 6:**
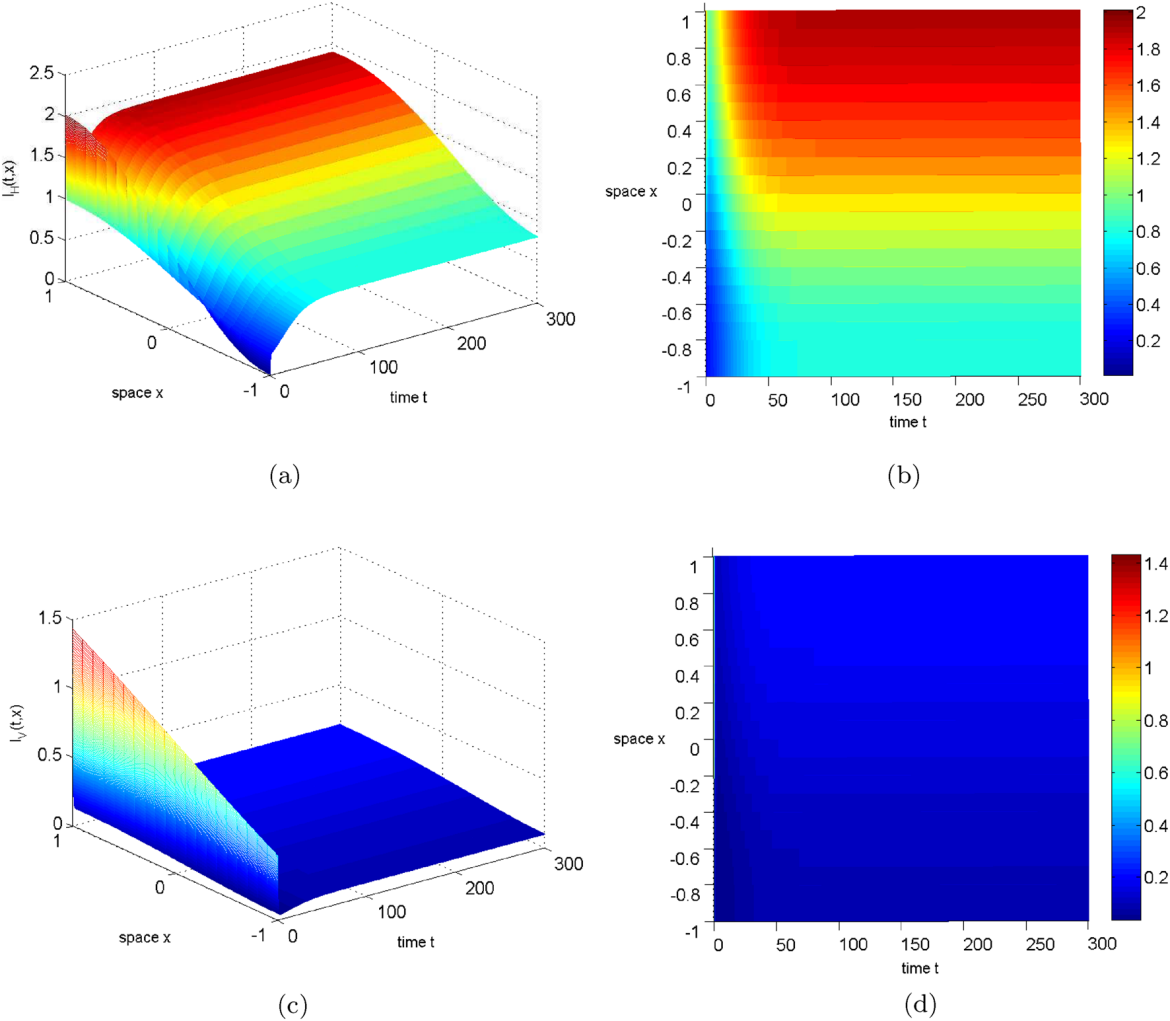
Numerical simulation of $I_{H},I_{V}$ for system (2) with $d_1=d_2=0.060$ (where $R_0=2.956695436468467>1$). Left: The evolution path of $I_{H},I_{V}$. Right: The distribution of $I_{H},I_{V}$ in time and space.

After the disease has gone extinct, the spread of humans and mosquitoes no longer affects its transmission. Thus, in this section we focus solely on the impact of diffusion on disease persistence, specifically on infected humans and mosquitoes. Figures  4, 5, and 6 reveal that increasing the diffusion coefficient reduces the infected area, but accentuates the spatial difference between infected humans and mosquitoes. This enhances disease persistence and disease control. Therefore, in the event of an outbreak, we recommend implementing appropriate measures to reduce the spread of humans and mosquitoes for more effective disease management.

## Conclusions

We conducted research on the threshold dynamics of a nonlocal diffusion dengue model with spatial heterogeneity. To establish the existence, uniqueness, positivity, and boundedness of the solution, we utilized the semigroup theory and the variation of constants formula. The expression of the basic reproduction number was abstractly determined using the next-generation matrix method. By constructing a Lyapunov function and applying the comparison principle, we proved the system’s global stability and uniform persistence. Numerical simulations were performed to verify the theorem. This study explored the evolution of disease extinction and persistence by adjusting the human transmission rate $\beta _{H}$. We also considered the impact of diffusion on infected humans and mosquitoes. The simulation results indicate that an increase in the diffusion coefficient leads to greater persistence of the disease in both humans and mosquitoes. This finding highlights the importance of controlling the spread of humans and mosquitoes during disease outbreaks. To achieve better disease control, we recommend implementing appropriate measures to reduce their transmission.

Additionally, we only researched on the threshold dynamics of a nonlocal diffusion dengue model. However, the transmission of dengue fever virus can also be affected by random factors, such as Lévy noise, Markov switching, etc. Therefore, it is interesting to introduce random noise into the nonlocal diffusion dengue fever model, at the same time, we will combine the stochastic nonstandard finite difference technique^29,30^ to make numerical calculations. Moreover, we note that the fractional derivative has been widely used in epidemiological studies^31–34^ due to its physical significance as a memory index. But in fact, for the model of nonlocal diffusion, since the nonlocal diffusion term is difficult to deal with, so we have not seen relevant research work. Next, we try to study the fractional order nonlocal diffusion dengue model.

## Data Availability

All data generated or analysed during this study are included in this published article.
